# Why Do Antibiotics Exist?

**DOI:** 10.1128/mBio.01966-21

**Published:** 2021-12-07

**Authors:** Fabrizio Spagnolo, Monica Trujillo, John J. Dennehy

**Affiliations:** a Biology Department, Queens College of The City University of New York, Flushing, New York, USA; b Department of Biological Sciences and Geology, Queensborough Community College, The City University of New York, Bayside, New York, USA; c The Graduate Center of The City University of New York, New York, New York, USA; Ohio State University

**Keywords:** antibiotic resistance, community assembly, cooperation, evolution

## Abstract

In the struggle with antibiotic resistance, we are losing. There is now a serious threat of moving into a postantibiotic world. High levels of resistance, in terms of both frequency and strength, have evolved against all clinically approved antibiotics worldwide. The usable life span of new clinically approved antibiotics is typically less than a decade before resistance reaches frequencies so high as to require only guarded usage. However, microbes have produced antibiotics for millennia without resistance becoming an existential issue. If resistance is the inevitable consequence of antibiotic usage, as has been the human experience, why has it not become an issue for microbes as well, especially since resistance genes are as prevalent in nature as the genes responsible for antibiotic production? Here, we ask how antibiotics can exist given the almost ubiquitous presence of resistance genes in the very microbes that have produced and used antibiotics since before humans walked the planet. We find that the context of both production and usage of antibiotics by microbes may be key to understanding how resistance is managed over time, with antibiotic synthesis and resistance existing in a paired relationship, much like a cipher and key, that impacts microbial community assembly. Finally, we put forward the cohesive, ecologically based “secret society” hypothesis to explain the longevity of antibiotics in nature.

## INTRODUCTION

## WHY DO ANTIBIOTICS EXIST IN NATURE?

**FIG 1:**
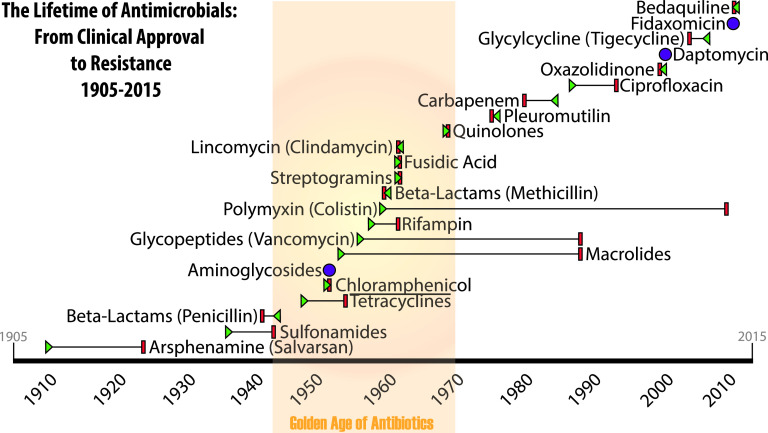
Antibiotic classes, their clinical introduction, and resistance identification. The resistance-free life spans of the major classes of antibiotics are indicated. Years of first clinical use (indicated by green triangles) reflect approval by regulatory agencies, usually in the United States. In most cases, the discovery of the class of compounds predates clinical approval by several years, although widespread use of the drug would not occur until approvals were secured. The year resistance was first reported in the literature is indicated by a red bar. In cases where resistance was reported prior to clinical approval, the red bar precedes the green triangle, which then points toward the red resistance bar. Cases where clinical approval was received in the same year that resistance was first reported are indicated by a purple circle. The antibiotic class with the longest resistance-free lifetime is polymyxin, which was not commonly used for decades due to toxicity in humans (108).

The majority of developed antibiotics owe their existence to microbes. Antibiotics likely have been part of the microbial world for millions of years (1, 2). But from the time they were discovered by humans and put into clinical use, we consistently see resistance evolve to high frequencies in short periods of time, typically within a decade or so (Fig. 1). Resistance is often to such a high degree, in terms of frequency and level, that the antibiotic becomes effectively useless for chemotherapeutic purposes. Given these two very different outcomes, we must consider why antibiotic use has not led to resistance in natural microbial usage as it so reliably has for humans.

The fact that all human use of antibiotics has led to antibiotic resistance implies that resistance may be inevitable. This outcome is not surprising given the intense selection for resistance in antibiotic-laden environments. But, if resistance is inevitable, why did resistance to all naturally occurring antibiotics not evolve and eliminate the possibility of miracle drugs long before the discovery of penicillin almost a century ago? Do microbes just manage antibiotics better, or is there something intrinsic about microbial use of these compounds that sustains their applicability? Why has resistance to even the oldest forms of naturally occurring antibiotics not reached fixation? Surely, enough time has elapsed for this possibility to occur. What do microbes and humans do differently? In short, we need to ask ourselves: why do antibiotics exist at all? Here, we review the current state of knowledge concerning antibiotic use and effect in both natural and human-controlled systems to better understand the role of antibiotics in the ecology and evolution of microbes past and present.

## THE ORIGIN OF ANTIBIOTICS AND THE ANTIBIOTIC ORIGIN STORY

The cornerstone of modern medicine was laid in the 1890s when Paul Ehrlich, working with *in vivo* stains, developed the idea of pairing stain-like compounds capable of entering cells with toxins that could kill it. Ehrlich’s work led to the development of salvarsan, the world’s first antibacterial (3). Dyes continued to be a focus in the new field of “chemiotherapy.” In 1935, Gerhard Domagk showed that prontosil, a red dye, was effective against streptococcal infections (4), making it the first sulfa drug (5). Prontosil was also highly effective against puerperal fever, most associated with deaths in women following childbirth (6). Sulfa drugs suffered from negative side effects and were used with limited success in the mid-twentieth century, particularly during World War II (7).

## ANTIBIOTICS

Salvarsan and prontosil were the flagship drugs of the first two classes of antimicrobial chemotherapy. But while bactericidal, they were both synthetic and not naturally occurring biomolecules. The first true antibiotic, penicillin, was discovered by Alexander Fleming in 1928. Fleming’s observation that a fungus, Penicillium notatum, inhibited the growth of staphylococci on agar medium led to the isolation and identification of what became known as penicillin.

Contrary to how we think of penicillin today, medicine was not revolutionized overnight, even though Fleming published his findings immediately (8). Rather, Fleming found that cultivating the fungus was difficult and that purifying penicillin was problematic. As such, what would later be called the miracle drug was not well known until the problem of production was taken up by Ernst Boris Chain, Howard Florey, Norman Heatley, and Edward Abraham. The work of these chemists led to the isolation and purification of penicillin along with techniques to efficiently prepare solutions that could then be administered to patients (9). Another milestone in the development of penicillin occurred when another fungal strain, Penicillium chrysogenum, which produced many times more penicillin than Fleming’s strain, was isolated from moldy fruit by Mary Hunt at the Northern Regional Research Laboratory in Illinois (10).

Clinical use of penicillin began in 1940, but the world’s first true antibiotic was largely ineffective against many infectious diseases because penicillin works only against Gram-positive bacteria (8). Treatment of Gram-negative bacteria would wait for the next major advancement in infectious disease control, which came when streptomycin was discovered in the lab of Selman Waksman at Rutgers University in 1943 (11).

## RISE OF RESISTANCE

The first antibiotic resistance described in published reports was to penicillin, in 1940 (12). Streptomycin resistance was reported in the first randomized controlled clinical trial for a drug in 1948 (13). In fact, resistance to all known antibiotics (whether natural or synthetic) followed quickly after clinical or industrial use of the drug (Fig. 1). The typical response was to identify and develop new antibiotics to replace older classes, leading to what has been called the golden age of antibiotics in the 1950s and 1960s (14). However, the pipeline of new, easily identified antibiotics was quickly exhausted (reviewed in reference 15), leading to a lack of available treatment options by the 1970s that continues today. Some programs, such as the 10 × ’20 initiative (16), have added newly approved antibiotics, but this trend seems likely to be short-lived (17).

There are two major reasons for the decline in new antibiotics over the past half-century: greater difficulty in identifying new classes of drugs (18) and the skyrocketing costs of development. The cost of bringing a new drug to market is estimated to be over $3.12 billion (converted from 2013 dollars) (19). With such an investment required, pharmaceutical companies have little financial interest in developing low-profit, short-treatment-duration medicines such as antibiotics. Financial concerns are further magnified given that the life expectancy of these new and expensive products has consistently been limited to less than a decade of clinical use due to antibiotic-resistant strains rising to high frequency within that time (Fig. 1).

In reaction to the antibiotic resistance problem and with treatment options dwindling, public health officials attempted to control antibiotic usage as a means of controlling resistance (20). The expectation was that rates of resistance would diminish as antibiotic usage dropped. However, resistance persisted even in the face of such drug management practices on both small and large scales (20–23). This failed approach for controlling the evolution of resistance by limiting usage was aptly named “the ecological fallacy” (21).

This fallacy is based upon a simplified view of evolution in which fitness gains due to antibiotic resistance are offset by fitness costs to bearers of resistance phenotypes due to reduced performance in other aspects of their biology, i.e., trade-offs. The flaw in this reasoning is the assumption that the absence of an antibiotic from the environment has the same level of impact as, but opposite effect of, its presence. We have learned that reversion to sensitivity is neither an immediate nor necessary outcome of selection simply because a resistant pathogen is no longer in an antibiotic-laden environment (24, 25). The reasons for this relate to the lasting impact of antibiotic treatment upon microbes as well as changes at the genetic level that reconfigure the fitness landscape for resistant populations and make reversion less likely (26–28). In essence, the presence of clinical concentrations of antibiotics more strongly selects for resistance than the absence of antibiotics selects for reversion to antibiotic sensitivity.

## THE ENVIRONMENTAL RESISTOME

Microbes are among the oldest extant organisms and during their billions of years of existence have come into close contact with toxic molecules of all types. Microbial genomics has provided a wealth of data indicating that most bacterial genomes have genes that allow them to become resistant to one or more antibiotics. These data have led to the development of the concept of an “antibiotic resistome,” which includes all antibiotic resistance genes (ARGs) and their precursors in both pathogenic and nonpathogenic bacteria (29, 30).

Notably, ARGs exist in all ecological niches that harbor microbial communities. These findings reinforce the essential role that antibiotics and other toxic substances have played in bacterial evolution and strongly suggest that resistance to antibiotics has coevolved along with antibiotic biosynthesis (reviewed in reference 31). The metagenomic analysis of ancient DNA shows clearly that antibiotic resistance is a natural mechanism that predates the use of antibiotics as therapeutic drugs (32). Soil bacteria play an essential role in the production of antibiotics and provide deep insight into the capacity to inactivate them. For instance, the genome of soil actinomycetes contains more than 20 biosynthetic gene clusters for diverse bioactive compounds and ARGs (33). In addition, there are soil bacteria that can use antibiotics as their sole carbon source (34). A deep understanding of the functional role of both antibiotic biosynthesis and ARGs in the environment can shed light onto how to address the future use of antibiotics to fight bacterial infections.

## NATURAL RESISTANCE

Retrospective investigations into the existence of resistance in the time before the introduction of penicillin (the preantibiotic era [Box 1]) indicate that ARGs were not only present but also common in a wide range of bacterial species (35), particularly on plasmids (36, 37). While the number of such preantibiotic-era studies is limited, we see that resistance to naturally occurring antibiotics was common (38), with more than one ARG existing for natural antibiotics such as penicillin (35). The number and diversity of ARGs in the preantibiotic era are much greater than expected, and plasmids carrying these genes have long been horizontally transferred within and between species (35). These data suggest that antibiotic resistance has been in existence for as long as antibiotics themselves (2). Why then did widespread antibiotic resistance never arise and sweep to fixation over the millions of years of consistent use by microbes, though it so reliably and irreversibly evolves in treatment for modern pathogens?

BOX 1 ANTIBIOTIC RESISTANCE IN THE PREANTIBIOTIC ERA

Bacteria are incredibly resilient organisms. Microbiology labs around the world regularly store bacterial samples for the long term by freezing a prepared sample in ultracold freezers. With a little care, these frozen bacterial samples can be viable again after thawing; in essence, the cells “wake up” and get back to growing and reproducing.

In times past, however, such storage techniques were not available to microbiologists. So how did these scientists store bacterial samples they collected? Many would store a collected sample in a stab tube. Stab tubes are sterile test tubes that have tightly fitting caps. These tubes can be filled with sterile nutrient agar, which solidifies at room temperature. The bacterial sample can then be stabbed into the agar and the test tube sealed and stored in the dark.

Storage in this manner regularly preserves bacteria for two to several years but in some cases even longer. From 1917 to 1954, bacteriologist E. G. D. Murray collected several hundred bacterial samples in this way. Because of his skilled sterile technique and careful practices, the Murray collection is still available for research and study. Most of the Murray collection predates the use of antibiotics and has served as an important research tool for understanding the types and levels of antibiotic resistance that existed in the preantibiotic era. Murray’s collection showed that low levels of antibiotic resistance were not uncommon and that plasmids containing antibiotic resistance genes predate clinical use of antibiotics. The Murray collection is now part of the National Collection of Type Cultures (NCTC), one of four major collections that make up Public Health England and supports researchers and clinicians worldwide.

## HOW ARE ANTIBIOTICS USED IN NATURE?

In nature, antibiotics are employed in a very different way than they are in clinical treatment. Most glaringly, antibiotics are not produced in such high concentrations, for such long durations, or at such large scales. In the clinic, antibiotics are used to kill all susceptible bacteria; any outside consequences of that use are ancillary. As such, the differences in the application of antibiotics between microbes and the clinic should be carefully considered, as these differences suggest that their effects may also differ.

## CONCENTRATION, DURATION, AND SCALE

Clinical use of antibiotics in humans is centered upon concentrated delivery of the compound over time. This usage directly ties concentration and duration together in a fundamental way. Clinically effective doses of antibiotics are identified using pharmacokinetics and pharmacodynamics (PK/PD [Box 2]). Dosing of an antibiotic to cure an infectious disease is a determined function of the peak concentration of antibiotic (a proxy of bacterium-killing power) and the efficacy of a delivered dose over time, usually 24 h (a proxy measure of chemical stamina *in situ*) (39). Put simply, the measured ability of a single dose of an antibiotic to kill bacteria and persist at detectable concentrations is optimized by PK/PD for clinical use against a bacterial strain of specified susceptibility. If low-level resistance is found in a strain, higher peak and residual concentrations over the specified time are used (39).

BOX 2 PK/PD BASICS

Antibiotics are known to work in the lab because we can measure things such as minimum inhibitory concentration (MIC) or the size of the zone of inhibition. But how do we go about determining what the dosage should be in the clinic? The answer to this question is pharmacokinetics and pharmacodynamics (PK/PD). In order to effectively and consistently kill a pathogen within a patient undergoing treatment, the concentration of the antibiotic must remain at or above the MIC for the pathogen. However, the concentration does not remain constant after dosing; it quickly climbs to a peak, known as the maximum concentration in serum (*C*_max_), and then drops off over some amount of time. A second dose is then needed to bring the concentration back up again before it falls below the specified MIC for the pathogen.

Plotting the serum concentration of the antibiotic over time produces a graph with a peak at *C*_max_, along with other information relevant to PK/PD analyses. For instance, the area under the concentration-time curve (AUC) for a specified time (usually 24 h) is a measure of how efficient each dose of antibiotic can be. Then, by normalizing *C*_max_ and AUC_24_ by MIC, a PK/PD “index” can be generated for a specific antibiotic-pathogen pairing.

PK/PD curves must be determined for humans and animals, since biologically active hosts will metabolize and excrete the antibiotic (40). This degradation reduces the total drug present over time, with the entire host environment capable of metabolizing the drug. Contrast this with natural environments where antibiotic concentrations will be more variable over time. Clearly there will be no PK/PD-like optimization and, in an environment such as soil, metabolic degradation will be more transient than would be the case in animal tissue. As a result, antibiotics in natural environments, such as soil, may linger for much longer periods of time than in clinical use. Experimental data support this conclusion (41), showing that antibiotics in soil, water, and even plants seem to persist over longer periods of time before they are degraded by biotic and abiotic reactions, mostly by microbial populations (42). But the ability of microbes to degrade environmental antibiotics suggests that this process is heavily dependent upon the type and size of microbial communities present, as well as abiotic conditions such as temperature, moisture, and pH (43). Given the reliance on such diverse (and uncontrolled) variables in the environment, antibiotic degradation is decidedly slow and heterogeneous. The most likely result is longer antibiotic residence time in the environment.

Concentration, however, is a different issue. There are no consistent data on natural antibiotic concentrations in the environment (reviewed in reference 42), but concentrations are reasonably suspected to be much lower than those used in the clinic (41, 44). For a compound to be functional as an antibiotic, i.e., either bactericidal or bacteriostatic, concentrations in nature must meet or exceed the MIC for the target species/strain. Often, the MIC can be extremely low (<0.016 mg/L or <0.016 ng/μL) (45). Such concentrations should be achievable in naturally growing populations, although likely over only limited distances. The result is that a single microbe can produce a small amount of an antimicrobial compound that raises the concentrations to or above MICs in the microliter-sized space around that particular cell. As many cells within the population produce the antibiotic, the concentration in the volumetric space around that population increases.

Nonetheless, concentrations of antibiotics in naturally occurring populations are not likely to approach even the same order of magnitude as in the clinic (46). Consider streptomycin: in patients with tuberculosis, target serum concentrations are often about 40 μg/mL (47), or 160× higher than the measured MIC for Mycobacterium tuberculosis (48) (and 2,500× higher than an MIC of 0.016 mg/L). This difference in concentration represents a drastic change in the magnitude of selection, with all low-level-resistant mutants being impacted in the same way and to the same degree as completely sensitive wild-type clones.

**FIG 2:**
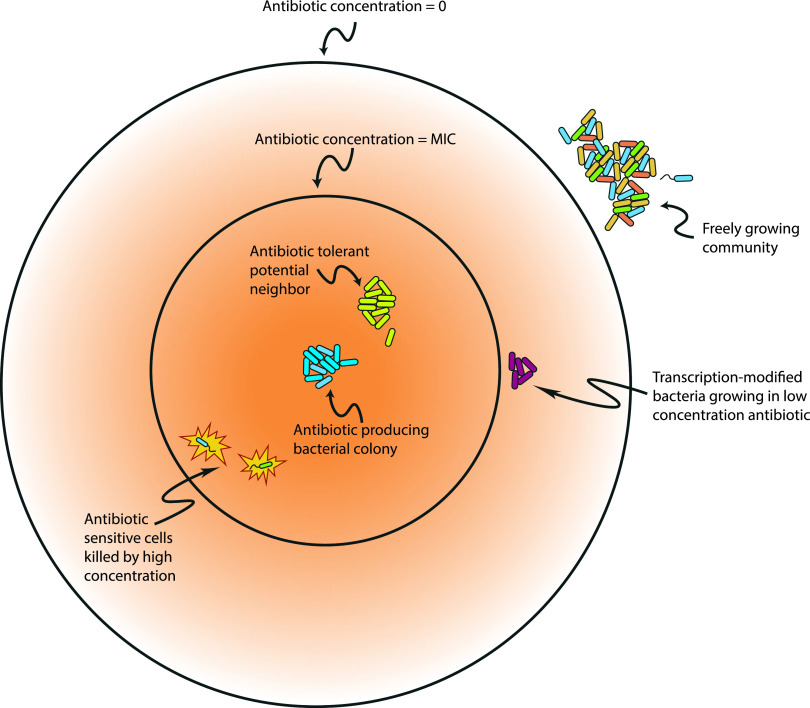
Effect of antibiotics upon microbial community interactions. In nature, antibiotics can have significant impacts upon interactions between species and strains of microbes. Rather than acting solely as a microbial weapon of warfare, we know that antibiotics can also function as signaling molecules and transcriptional modifiers. Antibiotic compounds can also create opportunities for community mutualisms with strains both resistant and sensitive, depending upon the context of the interaction. This suggests that antibiotics can play important and complex roles in community assembly and diversity and further implies the importance of paired production and resistance genes in microbial ecology.

The conclusion is that concentrations of naturally occurring antibiotic compounds, and their effects, are orders of magnitude lower than those used in the clinic. In addition, the time in which antibiotics remain in natural environments is different than in human patients (43). Pharmacodynamics not only optimizes peak concentrations but also sets lower limits below which serum concentration should not fall. This lower limit is often associated with the measured MIC for the pathogen. Any time spent below this level is implicated in selection for resistance mutations, such as in the mutant selection window hypothesis (49, 50). Therefore, from an evolutionary perspective, the way we use antibiotics is central to the current problem of antibiotic resistance and may be crucial to understanding how microbes have been able to employ antibiotics over long periods. In the natural situation described, a concentration gradient exists in space, with higher concentrations closer to the source and lower concentrations further away (Fig. 2). In the clinical picture, the concentration gradient exists in time, with values during treatment in a constant, cyclical, and controlled flux. In addition, antibiotic concentrations are roughly the same everywhere within the host at any given time. The important distinction between these two lies in the possible responses: a physical gradient can be tolerated through movement, perhaps even small ones. In contrast, a temporal flux, particularly at the concentrations considered, becomes an existential condition, necessarily impacting the ability to survive, and with it, the strength and direction of selection.

Similarly, it has been shown that combinations of antibiotics can enhance or diminish bacteriostatic or bactericidal effects (44, 51, 52), suggesting that clinical-level concentrations are not the only way of ensuring population-level effects upon microbes. This range of combinatorial effects adds depth to the number of possible outcomes in a localized environment as synergistic effects of antibiotics can both lower the probability of evolving resistance and expand the mutant selection window via antagonistic interactions.

The last of our immediate considerations is that of scale. The limited concentration and unknown duration of antibiotic compounds in natural environments contrast with the consistently high concentrations called for by PK/PD. Additionally, the impact of human use of antibiotics is global, in all senses of the word.

Within patients, antibiotics are distributed by design throughout the body. Ease of infection site delivery has always been a key advantage of antibiotics. Patients are treated with antibiotics orally, intravenously, rectally, or via injection, with circulatory systems facilitating transport to infected areas. This effortlessness is due in large part to the small size of antibiotic molecules and the fact that they have little cross-reactivity with molecules outside the target species (5). Contrast this with the complications of infection site delivery associated with other approaches, such as phage therapy (53, 54). The combination of the ease of delivery with the widespread use of broad-spectrum antibiotics to treat large groups of suspected (but not necessarily identified) pathogens results in the within-patient ubiquity of antibiotics upon which we have come to rely.

On a larger scale, since 1940, antibiotics have become an omnipresent pollutant in environments of all types (55). Even in places largely untouched by humans, we can find antibiotic compounds and, with them, ARGs (56–59). In recent years, we have learned that even low levels of antibiotic pollution select for high levels of resistance (60–62). Additionally, regardless of the biome or their ability to clear pollutants, humanity’s worldwide industrial-level use of antibiotics has resulted in a steady stream of antibiotics into the environment, without ever allowing natural systems to return to baseline levels (63).

The result of humanity’s overproduction and misuse of antibiotics, including their massive presence in agriculture and animal husbandry (64–66), is the transformation of a natural product used in limited scope within microbial communities into a biological weapon of mass destruction dispatched against microbial communities of all types in all places. This abuse is on the same scale as nuclear weapons testing, another misused mid-twentieth century technology (67). Although the expected duration of antibiotic contamination is thought to be significantly shorter than that of nuclear fallout (68), the long-term impact upon microbial communities and the genetic changes necessarily imposed by selection for resistance through contamination of the environment is beyond calculation. (Interestingly, although the timeline of nuclear fallout and its impact is much longer than that of antibiotic pollution in the environment, antibiotic pollution has already lasted longer in terms of generations for microbes. Radionuclides resulting from test explosions, such as plutonium-239 [half-life, 24,000 years], degrade on a scale of approximately 1,000 human generations. Even if we require several half-lives to have passed in order to accept a lower nuclear pollution level, that time is still dwarfed by the number of microbial generations that have passed since the industrial production of antibiotics started in 1942.)

## SIGNALING

If the concentration, duration, and scale of antibiotic use in nature are so vastly different from clinical use, the question of what need antibiotics evolved to meet remains. What is the role of antibiotics in natural environments? Potential answers to this question are few, but one hypothesis that has gained considerable support is that antibiotics function as a signaling molecules in microbial environments (69–71). The observation that low concentrations of antibiotic compounds can act as transcriptional regulators has become integral to our understanding of interspecific dynamics in natural microbial communities (72, 73). The transcriptional changes induced by antibiotic compounds range widely from species to species and compound to compound; however, many of the genes upregulated are implicated in stress response or biofilm-associated phenotypes, suggesting that the use of antibiotics in nature differs from their presumed role as weapons of microbial warfare. In fact, we argue that this presumption cannot be correct.

Consider how antibiotics are dispersed through space in a natural medium, such as soil. Microbial cells produce and excrete the antibiotic into the space around them. The concentration is highest at the source and drops with distance from the producer cells. The result is a zone of inhibition, the phenomenon Fleming first observed in 1928. A standardized paper disk or Kirby-Bauer test (74) is based upon the principle that the outer circumference of the zone of inhibition is directly related to the MIC for the bacterial strain tested, meaning that at the outer edge, the concentration of the antibiotic being tested is high enough to stop growth. However, the diffusion of the antibiotic can reasonably be expected to continue past the outer ring of the inhibition zone (disregarding any metabolic inactivation) and should continue to decrease over space, potentially affecting transcription in the bacterial cells present in this space (75). Contrast this with the PK/PD approach, where the goal is to maintain high serum concentrations in the entire volume over a specified time. Hormesis and the totality of antibiotic effects upon microbial populations are still not well understood (76). Combinations of antibiotics can also shift the effects of antibiotic compounds in an environment (44), suggesting that the lower concentrations found in nature might have oversized effects upon bacterial populations based on the totality of compounds present. Such synergistic or antagonistic effects may also inform hypotheses as to why microbes produce and excrete both bacteriostatic and bactericidal compounds, as well as offering a hypothesis underlying observations of antagonism between these different types of compounds, particularly at microbial scales (77).

In addition, the range of natural environments in animal hosts for microbes is large. Consider, for example, the different environments possible for “gut microbiomes.” In each of those, different bacterial communities with specific functional requirements will coexist. Furthermore, the impact of antibiotics on such communities (and their residual influence on host health) are vital to a complete understanding of the effect of antibiotics and resistance in natural microbial environments (78). Clearly, the use of antibiotics in natural environments has more to teach us about how and why these compounds have been in use for eons, forcing us to re-evaluate our assumption that the sole purpose of antibiotics lies in antagonism.

## REINTERPRETING ANTIBIOTICS WITH NATURE AS OUR ROSETTA STONE

Clinical use of antibiotics is rooted in the assumption that species producing a particular antibiotic do so for the purposes of directly competing against other species or strains in the natural environment. This presumption is the same as that which underlies antibiotic use by humans; antibiotics are armaments of antagonism, produced by one microbe and meant to hinder another.

Interestingly, humans depart from this supposition more than we adhere to it: clinical therapeutics is not the largest use of antibiotics and has not been for some time. In the 1940s, it was observed that livestock more efficiently achieved market size when given low doses of antibiotics (79, 80). While hypotheses for this observation exist (81), a complete explanation has not yet been deciphered. Nonetheless, we continue to use most industrially produced antibiotics not for microbial antagonism *per se* but rather for faster animal growth, without regard for the mode of action or its consequences.

Given humanity’s incomplete and inconsistent consideration of antibiotics, their application, and their purpose, a more complete analysis of their use under conditions shaped by evolutionary and ecological forces is warranted, particularly given the long success microbes have had using antibiotics and, perhaps more importantly, managing antibiotic resistance.

First and foremost, we note that, in nature, synthesis of and resistance to antibiotics are intimately connected. Studies of naturally occurring resistance indicate that an ARG is often linked with the genes involved in biosynthesis of that antibiotic (82). This association is confirmed in samples representing preantibiotic-era strains as well as those from pristine environments, with many of the antibiotic-resistance pairs encoded in phylogenetically related groups (38). The consistent pairing of antibiotic production with ARGs within monophyletic groups, coupled with such production-resistance pairings on mobile plasmids (35), strongly suggests that this connection signals a longstanding fundamental characteristic of antibiotic use in nature.

Phylogenetic ties between strains and groups are also supported by ecological interactions between and within these groups. More often than expected by a null model, microbial communities are made up of phylogenetically related interacting species (83, 84). The hypothesis underlying this observation is that abiotic variables in the environment filter community members and more closely related species are more likely to have similar niches (84, 85). We propose another possible hypothesis for this observation: community assembly is governed by biotic or, perhaps more properly, antibiotic filters as much as abiotic filters. This hypothesis explains the phylogenetic clustering observed in microbial communities across a large array of environments, as well as the consistency of groupings at the family taxonomic level observed in communities (83). Additionally, the existence of such mutualisms would also provide an explanation for the observations of antibiotic resistance arising as an emergent phenomenon within some communities as a result of cooperative action rather than from the biochemical processes within single cells (86).

As communities grow, distinct pairings that represent symbiotic interactions are often vital. It is beneficial for a species present to filter the pool of possible neighbors with which they will interact so as to increase the likelihood that a beneficial partner will be present, particularly when limited carbon resources are likely to be utilized by more than one member of the community (87). By producing and secreting a given antibiotic into the space around the producer’s position, a species may not be so much trying to impair competitors as to reserve space for proven partners. Experimental evidence of community construction based upon interactions in which antibiotics play a role has been observed (88, 89). Neighbors thus selected will need to be antibiotic resistant, which may also be more probable if they are also phylogenetically related to the producer. In addition, because of the pairing of antibiotic production genes with ARGs, the resistant neighbor is also more likely to be able to produce the same antibiotic compound, thereby reinforcing the screening effect and reserving even more space for the mutualistic pair to then grow into and/or control. Such a dynamic would indicate that resistance is precisely the mechanism that makes antibiotics valuable in natural microbial communities. Further, multimember community action is implicated as combinations of antibiotic-classes present can enhance or antagonize the effects of an antibiotic overall (52), leading to additional community-level filtering capacity.

The implication that natural antibiotic production and resistance should be intimately tied together can be contrasted with the presumption that antibiotics are a weapon of microbial warfare. In the combat framework, antibiotic production is beneficial, with the ability to produce more being better (akin to the PK/PD approach). This dynamic implies the strong potential for an evolutionary arms race. However, this system seems never to have dwelled in the realm of the red queen: hyperproducers (on the scale of clinical concentrations) are not known, nor are high levels of resistance. The strain of P. chrysogenum that Mary Hunt isolated from a rotting cantaloupe was a hyperproducer of penicillin, but artificial selection and X-ray mutagenesis experiments yielded even higher-producing mutants (90), suggesting that such a mutational path was possible via one or a few mutational steps but not likely to have ever been beneficial to fitness.

In addition, investigations into the evolution and long-term application of antibiotics as weapons of microbial warfare suggest that the microbial armory is large and diverse (91) but that evolutionary strategies that employ competition and antagonism ultimately need to be tightly controlled (92). Control is ultimately both costly and complex (92), particularly when eukaryotes are also considered (93). The cost of control mechanisms, along with their near-ubiquity across microbial species (92), suggest that a large-scale evolutionary arms race(s) was limited, even though the number and types of microbial “weapons” continued to diversify. A slightly different approach, and one that is in line with evolutionary game theory (94), is that even limited cooperation can be less costly over the number of interactions likely as well as over the evolutionary time scales involved. Consider that clinically relevant antibiotic resistance of the kind common today is known to be costly to fitness overall, making both sides of this evolutionary war game losing strategies without a consistent and strong offsetting force. Any breakdown of such a compensating tension would likely lead to antibiotics rapidly losing all potential benefit and being selected against by natural selection, followed by costly ARGs (although this argument runs dangerously close to replaying the ecological fallacy). Pairing these phenotypes, however, creates a balanced approach that can be maintained via selection by the constant need for community assembly across many environments over vast evolutionary timescales.

Even strains that are not resistant to a particular antibiotic may be influenced by low concentrations of the compound through transcriptional changes and gene regulation (95, 96). Genes that are transcriptionally impacted could possibly make the species more likely to cooperate with the antibiotic producer or, alternatively, less likely to invade the producer’s space (edge-specialist bacteria?). Such suppositions are amenable to experimental inquiry and offer one potential line of hypothesis testing and/or development.

This cooperative effect would presumably be greatest just beyond the outer edge of a zone of inhibition, where concentrations are lower, suggesting a kind of secret “friend or foe?” inquiry being put to a potential neighbor by the producer prior to any direct interaction. Similar systems are known to exist in quorum sensing feedbacks in species such as Clostridioides (formerly *Clostridium*) difficile (97), suggesting that nonconstitutive phenotypes can be induced based upon biointeractions with adjoining bacterial cells. Given the metabolic and fitness costs associated with antibiotic production, it is not surprising that researchers are also finding antibiotic synthesis linked to quorum sensing in cocultures (98, 99), particularly as a mechanism to limit social cheaters (100), suggesting that a main outcome of these interactions is related to community building and stability (101).

This “secret society” hypothesis, where known members are trusted, new members undergo hazing-like phenotype acquisition, and potential newcomers are continuously recruited, also provides a potential explanation for why ARGs in the clinic are often associated with plasmids. Preantibiotic-era plasmids often maintained a producer-resistance pairing. This may have been beneficial in increasing the likelihood of a pair of successful community members becoming neighbors again in another community even if they are not phylogenetically related. The probability of a mutualistic association could be increased in the future by the sharing of a plasmid by a producer: in essence, an initiation ritual. A recent study showed increased probability of plasmid persistence with increasing numbers of strains in a community (102). A similar system exists in colicinogenic plasmids in certain strains of Escherichia coli, where the colicin operon, containing a colicin-encoding gene (*cxa*), is bundled with the immunity-encoding gene (*cxi* or *imX*) (103). Plasmid transfer is also affected by antibiotic concentration, with the makeup and structure of microbial communities influencing what the target recipients of plasmids may be (104), suggesting again that antibiotics have long been tied to the structure and makeup of microbial communities.

If the secret society system was a dominant selective force in evolving and maintaining antibiotics in the preantibiotic era, then changes in the magnitude, frequency, and diversity of antibiotics after 1940 would likely have caused a consequent shift away from a production-resistance balance in favor of a resistance-dominated evolutionary strategy. We observe just such a signal when we compare the levels of resistance in preantibiotic era collections to modern circulating strains of bacteria (105). We also observe such co-option of existing plasmids in the environment (106). Such evidence compels us to consider the possibility that the propensity to donate a plasmid containing a production-resistance pair may have regularly been co-opted by bacteria after the dawn of the antibiotic age, driven by selection, as a measure to survive clinical treatment with high concentrations of industrially produced antibiotic compounds.

## GHOSTS OF RESISTOMES PAST

One potential concern inherent in a secret society system is that past violations in which the pairing was broken would likely already have become fixed within the relevant population(s). This leads to consideration of what potential signatures of the paired system might exist beyond those already mentioned.

The question of concern is whether broken pairings in the past could have led to fixation of ARGs, which would in turn lead to the eventual loss of synthesis genes for those specific natural antibiotics. If antibiotics were used in eons past not in a paired way but rather as a way to kill competitors, as has long been the presumption, then resistance to that antibiotic compound would be highly beneficial to fitness. The frequency of resistance carriers would increase and become fixed over time, rendering the antibiotic useless. The ability to synthesize the antibiotic would (presumably) be lost via selection over time. How, then, can we put forward the secret society hypothesis without being able to know if such a simple series of events happened in the distant past?

One possible approach would be to look for echoes of selection from long ago pairings compared to the natural antibiotic compounds present today. More precisely, we can look at the half-life of natural antibiotics in their relevant environments to understand how quickly they naturally degrade and use those data to inform a null hypothesis. There are three likely scenarios: extant natural antibiotics could have a longer half-life than other compounds, such as signaling molecules; they could have a shorter half-life; or they could have one that is about the same as that of other synthesized extracellular compounds.

Should natural antibiotics have a comparable half-life, not much new information can be gained. If they have a shorter half-life in the environment than similar extracellular molecules, that would imply that natural antibiotics are incapable of building up in the natural environment, rendering the likelihood of runaway selection for resistance low.

However, if the half-life of natural antibiotics in the environment is long, then selection for resistance to the accumulated concentration of these compounds becomes more likely. If this happened long ago, we would have lost any signal, because the resistance alleles would have become fixed perhaps before we even knew of microbes’ existence. Therefore, we would expect that natural antibiotics have a shorter half-life than most other naturally produced extracellular compounds, since we can still find such a diversity of them produced in microbial communities. While not a definitive test, such an approach provides for some baseline experimental inquiry.

## CONCLUSIONS AND FUTURE PROSPECTS

To have any hope of countering the rise in antibiotic-resistant infections, we will need to better understand the source, impact, and implications of the problem. While concern over infection rates is a biomedical matter, resistance is the result of evolutionary processes. Here, we argue that as a precursor to developing effective mitigation strategies, we need to expand consideration beyond either biomedicine or evolutionary biology and include the vast ecological dimension.

We began by asking why antibiotics exist at all, given that resistance to clinical drugs has so reliably evolved shortly after the introduction of every new class of antibiotics over the past 80 years. Antibiotics are not new; they are older than the human species. Yet, within a single human lifetime, many clinical antibiotics have been rendered ineffective. Within another lifetime, antibiotic resistance may become the scourge of our time. To understand this sea change, we look to the biological history of these secondary microbial metabolites for clues as to how their use might be preserved.

Most profound among the differences between the historic use by nature of antibiotics and their current therapeutic purpose is the divorce of antibiotic use from any counterbalanced resistance. Separating these ecologically conjoined twins and removing the context and countercontext put their trajectories on different, and ultimately dangerous, paths. The concentration, duration, and scale of human use created a potent artificial selection for antibiotic resistance to unnaturally high levels and frequencies. Evolution to resistance under clinical antibiotic conditions is, quite literally, a mandatory survival strategy for microbes.

Data collected over decades suggest that antibiotic production phenotypes have largely been paired with complementary antibiotic resistance phenotypes (2, 36, 37, 91). A similar pairing is found in other microbial toxin systems, such as in colicins (103). Nature’s signal in this regard seems clear: producing an antibiotic is not very meaningful without also being resistant. Horizontal gene transfer systems, particularly plasmids, reinforce this conclusion, but the number and type of such plasmids suggest that antibiotics play a larger role than just a weapon of war among microbes. We suggest that this larger role is one of mutualistic cooperation, particularly important in establishing and maintaining microbial communities.

Beneficial interactions in one community can become more likely in other communities if the interactors are both present in the new community. But we still do not understand all the ways in which cooperative interactions begin (107). Antibiotic use as described here may offer additional insight into this area. Novel, beneficial interactions can be reinforced and even reciprocated through production of an antibiotic by one or both participants. In addition, the exchange of antibiotic production and resistance genes could cement a relationship for future interactions. The importance of such mutualisms may be intensified given the structured spatial makeup of microbial communities, such as in biofilms, as well as the number and types of producers present (44, 51).

This pairing of production and resistance levels may have been the main means of maintenance over millions of generations. Neither production amounts nor resistance levels experienced directional selection pressure to increase or decrease, since there would be significant fitness costs without any benefit gained. In addition, if a given cooperation was beneficial to the community, that interaction would be selected for under the conditions experienced. In other words, there would exist selection for a maintained balance between antibiotic production and resistance.

Of course, the implication is that humans broke this microbial social contract by co-opting antibiotics under the presumption that they were an underutilized tool and transforming them into a biological weapon of mass destruction. In doing so, we created a potent selection for higher and higher resistance levels; losing the ability to control or manipulate it was inevitable, if not foreseen.

We have put forward the hypothesis that antibiotic resistance is not an evolutionary reaction to antibiotic warfare but rather a cocomponent of a preferred assembly mechanism for microbial communities. This hypothesis can be investigated experimentally. Such a series of experiments would continue to shine light not only on the mechanism and role of antibiotics in natural systems in the preantibiotic era but also on their importance in an ecological context. Finally, by understanding how the pairing of antibiotic production with resistance can achieve and maintain a balance, we will have developed the potential for mitigation strategies that may impact human health for lifetimes to come.
